# Evaluation of the Correlation between Regional Retinal Ganglion Cell Damage and Visual Field Sensitivity in Patients with Advanced Glaucoma

**DOI:** 10.3390/jcm11164880

**Published:** 2022-08-19

**Authors:** Amina Rezkallah, Ikrame Douma, Maxime Bonjour, Thibaud Mathis, Laurent Kodjikian, Philippe Denis

**Affiliations:** 1 Department of Ophthalmology, Croix-Rousse University Hospital, Hospices Civils de Lyon, University of Lyon, 69004 Lyon, France; 2 Department of Statistics, Hospices Civils de Lyon, University of Lyon, 69001 Lyon, France; 3 UMR-CNRS 5510 Matéis, University of Medicine Lyon 1, 69100 Villeurbanne, France

**Keywords:** glaucoma, optical coherence tomography, retinal ganglion cell layer, visual field, standard automated perimetry, structure-function correlation

## Abstract

(1) Background: to investigate the correlation between structural (retinal ganglion cells and retinal nerve fibers) and functional alterations analyzed point-by-point in the central 10 degrees of the visual field of patients with advanced glaucoma using Humphrey 10-2 visual field tests. (2) Methods: Single-center prospective cohort study carried on from October 2018 to February 2019 at the Croix-Rousse hospital, Lyon, France. The primary outcome measure was the point-by-point correlation between retinal sensitivity (Humphrey 10-2) and retinal ganglion cell complex (GCC) thickness. (3) Results: 29 eyes of 27 patients were examined. Of these, 15 eyes had a mean deviation (MD) less than −20 dB. There were statistically significant linear relationships between GCC thickness and 10-2 visual field sensitivity for several points in the lower part of the visual field, with lower retinal sensitivity being associated with thicker GCC layers. There were no strong linear relationships or statistically significant correlations in the other regions of the visual field. For the patients with MD < −20 dB, there were statistically significant linear relationships between GCC thickness and 10-2 visual field sensitivity for several points in the superior nasal region. Retinal sensitivity was not correlated with retinal nerve fibre layer thickness. (4) Conclusions: In this study of patients with advanced glaucoma, GCC thickness was linearly associated with 10-2 visual field sensitivity in certain regions, negatively for patients with less-severe glaucoma. The initial thickening raises questions about the apoptosis mechanism, while the thinning observed in the most severe cases is consistent with the ganglion cell death identified on visual field tests.

## 1. Introduction

Glaucoma is a progressive optic neuropathy that leads to more- or less-rapid visual field loss through retinal ganglion cell (RGC) and axon degeneration in excess of physiological age-related changes and in the absence of any ocular or neurodegenerative pathologies [1]. Ocular hypertension is the main and only modifiable risk factor [2,3,4,5].

The disease remains asymptomatic for a long time and functional signs only appear at a very advanced stage. Glaucoma is diagnosed based on a series of clinical and paraclinical signs. Systematic eye examinations are performed. Techniques are being developed to count and monitor RGCs in vivo [6]. In the meantime, glaucoma-related structural changes can be evaluated using fundus examinations and photographs of the optic nerve, but also using imaging techniques such as optical coherence tomography (OCT). The latter allows for objective measurements of optic disc size as well as peripapillary retinal nerve fibre layer (RNFL) and macular retinal layer thickness [7,8]. For several years now, the established method to evaluate functional changes is standard automated perimetry [9]. Nevertheless, testing patterns and strategies are still being developed to improve the speed, reliability, reproducibility and performance of the tests, and a firm structure–function relationship has yet to be established.

Several aspects of the measurement of this correlation have been studied to clarify the relationship and optimize the detection and monitoring of glaucoma. While it is clear the correlation exists, it manifests differently at different stages of the disease. At early stages, structural changes usually precede functional effects. In severe glaucoma, the damage seems to manifest as gradual visual field loss [10].

The aim of this study was to investigate the correlation between structural (ganglion cell complex (GCC, the layer of RGCs and the internal plexiform layer) and RNFL) and functional changes for each point in the central 10 degrees of the visual field (Humphrey 10-2 test) of patients with advanced glaucoma.

## 2. Materials and Methods

This was a single-center prospective cohort study of consecutive patients treated from October 2018 to February 2019 at the Croix-Rousse hospital in Lyon, France. The inclusion criteria were age ≥ 18 years, written informed consent, severe to advanced glaucoma (mean deviation (MD) worse than −12 dB according to the Hodapp, Parish and Anderson classification [11]), ability to undergo a reliable visual field test, best corrected visual acuity of at least 20/40, spherical equivalent of −5.00 to +4.00 dioptres. The corresponding exclusion criteria were: age <18 years; inability or unwillingness to provide informed consent; unwillingness to participate in the study; spherical equivalent <−5.00 D or >+4.00 D; previous eye surgery (except for uncomplicated cataract surgery more than 1 month previously and glaucoma surgery more than 3 months previously); a maculopathy or a neuropathy or neurological pathology affecting the visual field; inability to undergo examinations because of dementia or a cognitive, attention or psychiatric disorder; and eye disorders, namely dense cataracts, corneal dystrophy or degeneration affecting the visual field and tomography scans. The secondary exclusion criteria were poor tomography data (signal strength below 6/10, segmentation errors that could not be corrected manually) and uninterpretable visual field test results (false positives exceeding 15%, false negatives exceeding 33% and fixation losses exceeding 20%).

The demographic data recorded were the patients’ sex, age and ethnicity. The patients’ eye medical history and glaucoma severity were also recorded. White-on-white visual field tests were performed using a Humphrey analyser (Zeiss Humphrey System, Dublin, CA, USA) with standard settings (background luminance, 31.5 apostilb). The parameters recorded were the mean deviation (MD), pattern standard deviation (PSD), visual field index (VFI), and the retinal sensitivity for each point in the central 10 degrees (10-2 SITA standard program).

Ophthalmological examinations included measurements of best corrected visual acuity, intraocular pressure using a Goldman applanation tonometer, central corneal thickness by ultrasound pachymetry (OcuScan^®^ RxP Ophthalmic Ultrasound System Pachymeter, Alcon, Geneva, Switzerland), the axial length of the eye by optical biometry (IOLMaster 500, Zeiss Medical Technology, Dublin, CA, USA) and the iridocorneal angle by static or dynamic gonioscopy. The anterior segment of the eye was examined using a slit lamp and the fundus using an ophthalmoscope.

Optical coherence tomography (Angioplex Cirrus, Carl Zeiss Medical Technology, Dublin, CA, USA) was used to investigate retinal nerve fibres and GCC thickness. The latter was measured in a 6 mm × 6 mm area of the macula with automatic segmentation using the Cirrus OCT ganglion cell analysis algorithm (v. 6.0), which estimates the combined thickness of the RGCs and the internal plexiform layer by identifying the external limits of the RNFL and of the internal plexiform layer. The mean and minimal thicknesses were recorded along with the thickness in the superior temporal, superior, superior nasal, inferior nasal, inferior and inferior temporal regions. Tomography scan results were excluded if the signal strength was less than 6.

The primary outcome measure was the correlation between GCC thickness and retinal sensitivity for each point of the 10-2 visual field.

**Figure 1:**
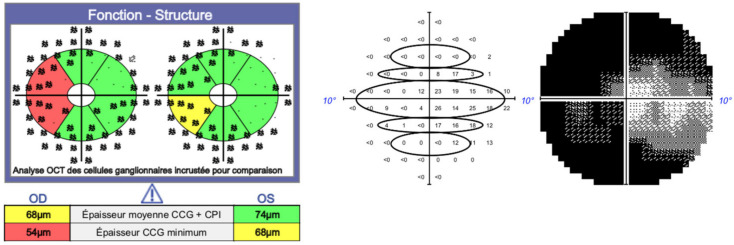
Investigation of structure–function correlations using the software GPA II and Cirrus. The visual field testing areas have to be moved to overlap with the corresponding areas in the ganglion cell maps, as described by Hood and Raza. The black circles show the points of sensitivity in the 10-2 visual field map we used for the analysis. OCT: ocular coherence tomography; OD: right eye; OS: left eye; CCG: ganglion cell complex; CPI: inner plexiform layer.

The secondary outcome measures were the correlations between RNFL thickness and retinal sensitivity in the inferior and superior regions and the correlation between GCC and retinal sensitivity specifically for patients with MDs worse than −20 dB, evaluated in both cases for all the points in the 10-2 visual field map. Correlations were investigated using the software GPA II (Carl Zeiss Medical Technology, Dublin, CA, USA), as illustrated in Figure 1.

All analyses were performed using the software R, version 3.5.2. (Microsoft, Redmond, Washington, DC, USA) Quantitative variables were described by the mean and range of the distribution and categorical variables were described as the frequency and percentage in each category. The associations between structure and function variables were estimated using linear and logarithmic regression analysis. The results were summarized as the slope of the regression line (the strength of the linear relationship), the coefficient of determination (R^2^) and the *p*-value of the regression slope. Results were considered statistically significant at *p* ≤ 0.05. No correction for multiple comparisons was applied, this being a preliminary descriptive study.

## 3. Results

### 3.1. Study Population

A total of 30 eyes (28 patients) were included in the study. One patient retracted consent, so the final study group consisted of 27 patients (29 eyes, 16 right). The patients’ mean age was 65 years (20–89 years), and 10 (37%) were women. Glaucoma was classified as primary open-angle for 20 eyes and chronic angle closure glaucoma for 5 eyes. There was one case of juvenile glaucoma, two of pigmentary glaucoma and one of pseudoexfoliative glaucoma.

The mean size of the optic nerve was 1.9 mm (0.8–2.2), with a mean cup-to-disc ratio of 0.85 (0.60–0.99). The mean corneal thickness was 505 µm (418–581 µm). The mean values of the RNFL thickness, GCC thickness and MD were 56.6 µm (42–74 µm), 59 µm (38–98 µm) and −17.7 dB (−30.5 to −13.9 dB), respectively. A total of 15 eyes (52%) had a MD worse than −20 dB.

### 3.2. Correlation between GCC Thickness and Retinal Sensitivity

**Figure 2:**
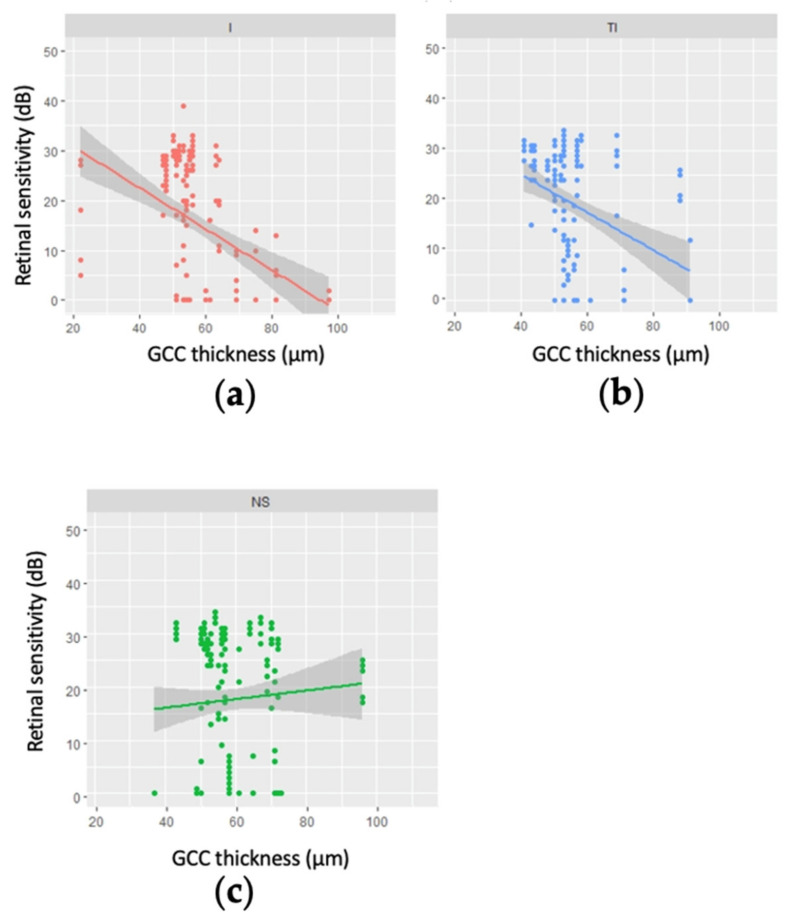
Linear regression modelling of retinal sensitivity as a function of ganglion cell complex (GCC) thickness for regions of the 10-2 visual field for 27 patients (29 eyes) with advanced glaucoma. (**a**) the inferior region—the regression slopes ranged from −0.48 to −0.33 (*p* = 0.01–0.09); (**b**) the inferior temporal region—the regression slopes ranged from −0.37 to −0.21 (*p* = 0.05–0.19); (**c**) the nasal superior region—none of the regression slopes were statistically significant (*p* > 0.3) and the coefficients of determination were low (R^2^ < 0.04).

Statistically significant linear relationships were identified between GCC thickness and retinal sensitivity for several points in the inferior and inferior temporal regions of the visual field (Figure 2 and online Supplementary Table S1). The regression slopes ranged from −0.48 to −0.33 in the inferior region (*p* = 0.01–0.09) and −0.37 to −0.21 in the inferior temporal region (*p* = 0.05–0.19), indicating that retinal sensitivity tended to be lower where the GCC was thicker. In logarithmic analysis (online Supplementary Table S2), the relationships were generally stronger, with slopes ranging from −4.01 to −3.08 in the inferior region (*p* = 0.01–0.05), −3.48 to −2.58 in the inferior temporal region (*p* = 0.02–0.15), and −2.48 to −1.01 in the superior temporal region (*p* = 0.05–0.58), with, once again, lower retinal sensitivities being associated with thicker GCCs. The opposite tendency was observed in the superior nasal region (Figure 2c and online Supplementary Tables S1 and S2), but none of the regression slopes were statistically significant (*p* > 0.3) and the coefficients of determination were low (R^2^ < 0.04).

**Figure 3:**
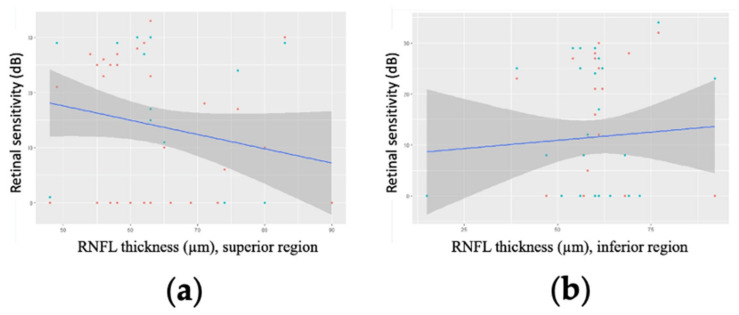
Linear regression modelling of retinal sensitivity as a function of mean retinal nerve fibre layer (RNFL) thickness for 27 patients (29 eyes) with advanced glaucoma for two points in (**a**) the superior part of the visual field (*p* = 0.12) and (**b**) the inferior part of the visual field (*p* = 0.62).

**Figure 4:**
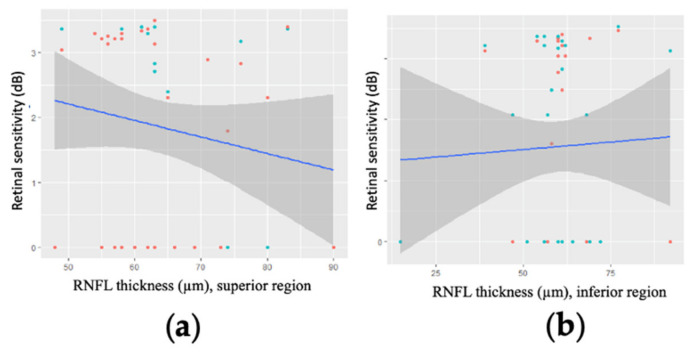
Logarithmic regression modelling of retinal sensitivity as a function of mean retinal nerve fibre layer (RNFL) thickness for 27 patients (29 eyes) with advanced glaucoma for two points in (**a**) the superior part of the visual field (*p* = 0.24) and (**b**) the inferior part of the visual field (*p* = 0.37).

No significant correlations were identified between mean RNFL thickness and retinal sensitivity, either in linear (Figure 3) or logarithmic analysis (Figure 4).

### 3.3. Correlation between Mean Regional GCC Thickness and Retinal Sensitivity for Patients with an MD Worse than −20 dB

**Figure 5:**
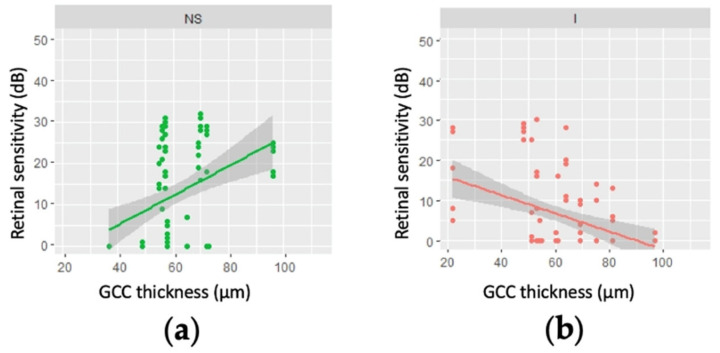
Linear regression modelling of retinal sensitivity as a function of ganglion cell complex (GCC) thickness for regions of the 10-2 visual field for 15 patients (15 eyes) with a mean deviation worse than −20 dB. (**a**) the nasal superior region—the regression slopes ranged from 0.40 to 0.49 (*p* = 0.15); (**b**) the inferior region—the regression slopes ranged from −0.31 to −1.08 (*p* = 0.17).

Considering only the 15 eyes with an MD worse than −20 dB, the relationships between GCC thickness and retinal sensitivity were of borderline significance (0.10 > *p* > 0.05) for several points in the inferior superior nasal regions of the visual field, in both linear (regression slopes, 0.40–0.49) and logarithmic analysis (regression slopes, 0.40–0.49 and 3.12–4.25, respectively; online Supplementary Tables S3 and S4). Figure 5 shows that while retinal sensitivity tended to be higher where the GCC was thicker in the superior nasal region (Figure 5a), thicker GCCs were associated with poorer retinal sensitivity in the inferior region (Figure 5b).

**Figure 6:**
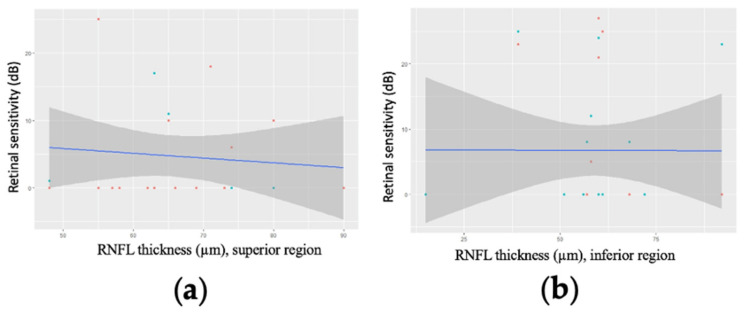
Linear regression modelling of retinal sensitivity as a function of mean retinal nerve fibre layer (RNFL) thickness in regions for two points of the 10-2 visual field for 15 patients (15 eyes) with a mean deviation worse than −20 dB. (**a**) the superior part of the visual field (*p* = 0.622); (**b**) the inferior part of the visual field (*p* = 0.989).

In patients with a MD worse than −20 dB, mean RNFL thickness was not correlated with retinal sensitivity in any part of the visual field, either in linear analysis (Figure 6) or after logarithmic transformation.

## 4. Discussion

In this study of patients with severe glaucoma, significant linear associations were identified in several parts of the visual field between GCC thickness and retinal sensitivity. In patients with less-advanced glaucoma, retinal sensitivity tended to decrease with increased GCC thickness in the inferior part of the visual field. For patients with an MD worse than −20 dB, the tendency was the same but weaker in the inferior area, but the retinal sensitivity increased with GCC thickness in the superior nasal area. This is the first time the correlation between GCC thickness and retinal sensitivity has been investigated point-by-point across the visual field.

Aptel et al. [12] identified statistically significant associations between retinal sensitivity and the RNFL thickness in several region of the visual field, the associations being strongest in the superior temporal area and weakest in the nasal area.

Around 30% of the RGCs damaged in glaucoma are in the macula [13]. The papillomacular bundle is more resilient to structural damage in glaucoma and changes in macular thickness are less likely to reach the measurement threshold than those in the RNFL are. Therefore, macular measurements are useful in patients with advanced glaucoma. Shin et al. have recently shown that RGC and GCC parameters are more responsive to changes in advanced glaucoma than RNFL monitoring is [14].

Very recently, Sung et al. found a weak correlation in patients with advanced glaucoma between RNFL thicknesses above the detection threshold and retinal sensitivity in 10-2 visual field tests [15]. Macular parameters (superior nasal internal plexiform layer thickness and internal and external nasal macular thickness) were also significantly correlated with visual field test data [15]. However, Sung et al. only considered the MD, PSD, and the foveal threshold and did not perform a point-by-point analysis [15]. No significant correlation was identified between RNFL parameters and 10-2 visual field test data, either in Sung et al.’s study or in ours. Elsewhere, Drasdo et al. have shown that the structure–function relationship is much less linear in the macular region because of lateral displacement of the RGCs [16].

Several factors influence the correspondence between structural and functional alterations in glaucoma. The use of different units—micrometers for thickness measurements and decibels for functional tests—leads to non-linear structure–function relationships [17]. Aptel et al. [12] and more recently Sung et al. [15] have shown that logarithmic fits of these data are more reliable than linear analyses. Comparing the thickness of ganglion cells with retinal sensitivity over areas corresponding to multiple points in the visual field may bias the correlation; hence, a more detailed map of the thickness of ganglion cells would be interesting.

Visual field maps have been shown to vary between individuals [18]. This variability stems mainly from interindividual differences in the positioning of the fovea with respect to the optic disc and the axial length and size of the optic disc [19]. One limitation of the study was its transversal design; a longitudinal design may compensate for the bias in the structure–function relationship induced by interindividual variability.

Optical coherence tomography is the most widely used technique for the early detection of structural abnormalities and to monitor glaucoma in terms of RNFL or GCC thinning [20,21,22,23]. In advanced glaucoma however, OCT cannot reliably measure tissue thickness, probably because there is a detection limit below which the technique is no longer sensitive to changes [24,25,26]. The sensitivity of OCT is limited by the presence of residual tissues such as glial cells and blood vessels [27] and by the performance of tissue segmentation algorithms. This detection limit is problematic for glaucoma monitoring, as anatomical changes below the threshold are invisible to optical imaging [28]. Histologically, indeed, non-neuronal elements such as blood vessels and glial cells can be included in RNFL thickness measurements along with RGCs. The non-neuronal elements can remain even after complete RGC loss as they do not degenerate simultaneously. Wang et al. have shown that glial cells can proliferate in a glaucomatous retina [29]. Nevertheless, Bowd et al. have shown that OCT measurements are sensitive to disease-related changes in advanced glaucoma and that anatomical monitoring remains worthwhile in advanced glaucoma [30].

The main limitation of this study is the low number of patients. However, although patient numbers are low, the structure–function correlation observed for patients with MDs worse than −20 dB differs from the one observed in patients with MDs of between −12 and −20 dB. In the patients with an MD <−20 dB, the strongest correlations were observed in the superior nasal region, where thinner GCCs were associated with poorer retinal sensitivity. These results are interesting and raise the question of the pathophysiological chronology of glaucoma. Several factors can lead to ganglion cell loss in glaucoma including axonal transport deficits, neurotrophic factor deficiencies, activation of intrinsic and extrinsic apoptotic signaling pathways, mitochondrial defects, excitotoxic damage, oxidative stress, loss of synaptic transmission and glial dysfunction [31]. Excessive glial activation involving astrocytes, Müller cells and microglia [29] is an important factor in the propagation of inflammatory processes and neurodegeneration in glaucoma. Retinal ganglion cell apoptosis is the main component of glaucomatous neuropathy. However, axonal or synaptic degeneration processes independent from the initial apoptotic process are also involved. Two axonal self-destruction processes have been described: Wallerian degeneration, a rapid, anterograde process that leads to the complete destruction of the axon and the synapse following acute stress, and retrograde degeneration, a deferred, progressive response to chronic stress. It may be that patients with severe glaucoma with a MD worse than −12 dB do not all have complete RGC loss. Since the apoptosis process first involves cell fragmentation, there is probably an initial phase in which the RGC layer does not decrease in thickness, while at the same time an inflammatory process mediated by glial and microglial activity increases RGC volume. In more advanced glaucoma however, this inflammation eventually leads to phagocytosis and cell loss. These results are preliminary and exploratory, and a longitudinal design and an increase of the population will help to confirm these proposed hypotheses.

While automated perimetry remains the main technique used to monitor glaucomatous neuropathy in patients with advanced glaucoma, retinal sensitivity measurements in visual field tests are variable, especially in more severe cases [32,33].

Many of the analyses in our study were performed on data from 29 eyes. It would be interesting to increase the sample size to confirm or correct the structure–function relationships described here and the corresponding pathophysiological explanations. Another limitation of the study was the detection limit of OCT; to overcome this, these analyses could be repeated in patients with mild and moderate glaucoma. Another avenue for future research is the possible regional correlations between vascular damage detected by OCT angiography, structural damage, and retinal sensitivity for each point in the visual field.

## 5. Conclusions

In this study of patients with advanced glaucoma, GCC thickness was linearly associated with 10-2 visual field sensitivity in certain regions, and negatively for patients with less-severe glaucoma. The initial thickening raises questions about the apoptosis mechanism, while the thinning observed in the most severe cases is consistent with the ganglion cell death identified on visual field tests.

## Data Availability

Not applicable.

## Supplementary Materials

The following supporting information can be downloaded at: https://www.mdpi.com/article/10.3390/jcm11164880/s1, Table S1: Linear regression parameters for retinal sensitivity as a function of ganglion cell complex thickness (in µm) for various points in the 10-2 visual of 27 patients (29 eyes) with advanced glaucoma; Table S2: Logarithmic regression parameters for retinal sensitivity as a function of ganglion cell complex thickness (in µm) for various points in the 10-2 visual field of 27 patients (29 eyes) with advanced glaucoma; Table S3 Linear regression parameters for retinal sensitivity as a function of ganglion cell complex thickness (in µm) for various points in the 10-2 visual field of 15 patients (15 eyes) with a mean deviation worse than −20 dB; Table S4: Logarithmic regression parameters for retinal sensitivity as a function of ganglion cell complex thickness (in µm) for various points in the 10-2 visual field of 15 patients (15 eyes) with a mean deviation worse than −20 dB.
