# Supplementary material for: Evaluation of the Correlation between Regional Retinal Ganglion Cell Damage and Visual Field Sensitivity in Patients with Advanced Glaucoma
Source: J Clin Med. 2022 Aug 19;11(16):4880. doi: 10.3390/jcm11164880 (PMC9409684; doi:10.3390/jcm11164880)
Supplement: Supplementary file 1 [file jcm-11-04880-s001.zip › jcm-1808912-supplementary.pdf]

Table S1. Linear regression parameters for retinal sensitivity as a function of ganglion cell complex thickness (in μm) for various points in the 10-2 visual field of 27 patients (29 eyes) with advanced glaucoma

Region	Regression equation	R^2	*p*
Superior nasal			
1	y = 0.09x + 56.91	–0.026	0.600
2	y = 0.1x + 56.9	–0.024	0.565
3	y = 0.1x + 56.86	–0.023	0.554
4	y = 0.02x + 58.36	–0.036	0.897
5	y = 0.07x + 57.56	–0.030	0.663
6	y = 0.01x + 58.93	–0.037	0.937
Superior			
1	y = 0.13x + 55.4	–0.022	0.531
2	y = –0.09x + 58.53	–0.030	0.679
3	y = 0.05x + 57.04	–0.035	0.817
4	y = –0.25x + 60.83	0.019	0.227
5	y = –0.26x + 60.63	0.023	0.208
6	y = –0.22x + 59.99	0.001	0.320
Superior temporal			
1	y = –0.18x + 59.45	0.006	0.287
2	y = –0.25x + 60.47	0.058	0.110
3	y = –0.25x + 60.36	0.072	0.087 *
4	y = –0.2x + 58.95	0.029	0.188
5	y = 0x + 55.86	–0.037	0.985
6	y = –0.16x + 57.9	0.006	0.288
Inferior nasal			
1	y = –0.18x + 61.01	0.014	0.248
2	y = –0.15x + 60.45	–0.001	0.331
3	y = –0.22x + 61.76	0.035	0.168
4	y = –0.2x + 61.21	0.020	0.222
5	y = –0.03x + 57.83	–0.036	0.881
6	y = –0.18x + 60.82	0.011	0.261
Inferior			
1	y = –0.33x + 62.66	0.071	0.088 *
2	y = –0.47x + 63.4	0.193	0.010 **
3	y = –0.33x + 61.63	0.072	0.086 *
4	y = –0.47x + 64.75	0.200	0.009 **
5	y = –0.42x + 62.52	0.136	0.028 **
6	y = –0.48x + 64	0.195	0.010 **

Inferior temporal			
1	y = –0.22x + 60.62	0.031	0.180
2	y = –0.31x + 62.16	0.108	0.046 **
3	y = –0.33x + 62.79	0.097	0.055 *
4	y = –0.21x + 59.91	0.027	0.194
5	y = –0.31x + 62.08	0.099	0.053 *
6	y = –0.37x + 63.44	0.128	0.032 **

*$p < 0.1$; **$p < 0.05$

Table S2. Logarithmic regression parameters for retinal sensitivity as a function of ganglion cell complex thickness (in μm) for various points in the 10−2 visual field of 27 patients (29 eyes) with advanced glaucoma

Region	Regression equation	R^2	*p*
Superior nasal			
1	y = 1.36x + 55.35	−0.009	0.395
2	y = 1.42x + 55.35	−0.005	0.364
3	y = 1.39x + 55.46	−0.005	0.358
4	y = 0.74x + 57.15	−0.028	0.628
5	y = 0.73x + 57.25	−0.026	0.592
6	y = 0.47x + 57.8	−0.033	0.735
Superior			
1	y = 1.27x + 54.83	−0.018	0.486
2	y = 0.34x + 57.03	−0.035	0.843
3	y = 1.07x + 56.01	−0.023	0.549
4	y = −0.82x + 59.03	−0.029	0.643
5	y = −2x + 60.71	0.019	0.226
6	y = −1.59x + 60.05	−0.005	0.360
Superior temporal			
1	y = −1.01x + 58.59	−0.025	0.577
2	y = −1.94x + 60.79	0.017	0.234
3	y = −2.48x + 61.54	0.109	0.045 **
4	y = −2.19x + 60.53	0.068	0.092 *
5	y = 1.02x + 53.86	−0.014	0.435
6	y = −1.23x + 58.06	0.001	0.322
Inferior nasal			
1	y = −1.73x + 61.61	0.021	0.248
2	y = −1.26x + 60.42	−0.006	0.331

3	y = −2.35x + 63.19	0.061	0.168
4	y = −1.92x + 61.97	0.032	0.222
5	y = 0.03x + 57.24	−0.037	0.881
6	y = −2.17x + 62.66	0.037	0.261
Inferior			
1	y = −3.06x + 63.93	0.072	0.072 *
2	y = −4.01x + 64.14	0.224	0.224
3	y = −3.69x + 64.41	0.134	0.134
4	y = −3.37x + 64.09	0.125	0.125
5	y = −3.08x + 62.52	0.106	0.106
6	y = −3.71x + 64.34	0.155	0.155
Inferior temporal			
1	y = −1.51x + 60	−0.005	0.360
2	y = −2.77x + 62.65	0.106	0.047 *
3	y = −2.96x + 63.67	0.097	0.056 *
4	y = −2.03x + 60.68	0.042	0.147
5	y = −2.59x + 62.29	0.083	0.071 *
6	y = −3.48x + 64.89	0.144	0.024 **

*$p < 0.1$; **$p < 0.05$

Correlation between mean regional RNFL thickness and retinal sensitivity

Table S3. Linear regression parameters for retinal sensitivity as a function of ganglion cell complex thickness (in µm) for various points in the 10-2 visual field of 15 patients (15 eyes) with a mean deviation worse than −20 dB

Region	Regression equation	R^2	*p*
Superior nasal			
1	y = 0.41x + 55.17	0.091	0.146
2	y = 0.49x + 54.62	0.151	0.085*
3	y = 0.4x + 55.76	0.086	0.151
4	y = 0.45x + 57.51	0.027	0.259
5	y = 0.46x + 55.33	0.131	0.101
6	y = 0.47x + 56.23	0.082	0.158
Superior			
1	y = 0.65x + 55.06	0.137	0.096*
2	y = 0.4x + 59.49	−0.021	0.415
3	y = 0.69x + 57.49	0.087	0.150
4	y = −0.1x + 62.86	−0.074	0.846
5	y = −0.16x + 63.22	−0.065	0.714
6	y = −0.14x + 63.06	−0.069	0.762
Superior temporal			
1	y = −0.31x + 61.89	−0.026	0.435
2	y = −0.54x + 63.76	0.112	0.120
3	y = −0.43x + 61.81	0.100	0.134
4	y = −0.44x + 61	0.058	0.196
5	y = 0.24x + 54.9	−0.039	0.504
6	y = −0.23x + 58.77	−0.035	0.482
Inferior nasal			
1	y = −0.14x + 61.04	−0.056	0.619
2	y = −0.1x + 60.54	−0.065	0.712
3	y = −0.21x + 62.07	−0.030	0.453
4	y = −0.18x + 61.33	−0.048	0.562
5	y = 0.11x + 57.69	−0.065	0.704
6	y = −0.16x + 60.9	−0.058	0.634
Inferior			
1	y = −0.31x + 63.04	−0.036	0.484
2	y = −1.08x + 65.5	0.245	0.035 **
3	y = −0.6x + 63.47	0.006	0.317
4	y = −0.73x + 66.97	0.191	0.059 *

5	y = −0.87x + 64.32	0.101	0.133
6	y = −0.69x + 65.46	0.144	0.090*
Inferior temporal			
1	y = −0.17x + 60.97	−0.056	0.621
2	y = −0.37x + 62.93	0.035	0.241
3	y = −0.34x + 63.74	0.027	0.259
4	y = −0.12x + 60.25	−0.065	0.706
5	y = −0.36x + 63.31	0.045	0.219
6	y = −0.35x + 63.63	0.025	0.266

*$p < 0.1$; **$p < 0.05$

Table S4. Logarithmic regression parameters for retinal sensitivity as a function of ganglion cell complex thickness (in µm) for various points in the 10-2 visual field of 15 patients (15 eyes) with a mean deviation worse than –20 dB

Region	Regression equation	R^2	*p*
Superior nasal			
1	y = 3.93x + 53.52	0.135	0.097*
2	y = 4.25x + 53.44	0.169	0.072*
3	y = 3.45x + 55.06	0.101	0.133
4	y = 3.12x + 56.72	0.028	0.256
5	y = 3.52x + 55.4	0.118	0.114
6	y = 3.57x + 55.68	0.101	0.132
Superior			
1	y = 4.9x + 54.28	0.122	0.109
2	y = 4.76x + 57	0.089	0.148
3	y = 5.58x + 56.01	0.154	0.082 *
4	y = 1.36x + 60.36	−0.065	0.709
5	y = −1.36x + 63.43	−0.063	0.681
6	y = −0.68x + 62.83	−0.074	0.842
Superior temporal			
1	y = −2.21x + 62.63	−0.053	0.599
2	y = −3.34x + 64.23	0.008	0.309
3	y = −3.45x + 62.42	0.116	0.116
4	y = −3.24x + 61.73	0.070	0.176
5	y = 2.86x + 52.66	0.031	0.251
6	y = −1.98x + 59.34	−0.026	0.437
Inferior nasal			
1	y = −1.37x + 61.49	−0.049	0.570
2	y = −0.69x + 60.34	−0.070	0.775
3	y = −2.26x + 63.15	−0.006	0.357
4	y = −1.67x + 61.9	−0.038	0.498
5	y = 1.37x + 56.67	−0.055	0.609
6	y = −2.05x + 62.6	−0.033	0.469
Inferior			
1	y = −2.28x + 63.66	−0.040	0.507
2	y = −6.79x + 65.47	0.225	0.042 **
3	y = −4.59x + 65.11	0.040	0.231
4	y = −3.85x + 65.54	0.048	0.214
5	y = −3.85x + 63.54	0.013	0.297
6	y = −4.57x + 65.7	0.079	0.162
Inferior temporal			

1	y = −0.12x + 59.35	−0.077	0.965
2	y = −2.63x + 62.88	0.019	0.280
3	y = −2.56x + 63.78	0.013	0.298
4	y = −1.3x + 60.74	−0.054	0.604
5	y = −2.31x + 62.68	−0.001	0.337
6	y = −3.09x + 64.68	0.05	0.210

$*p < 0.1$; $**p < 0.05$

Correlation between mean regional RNFL thickness and retinal sensitivity for patients with a MD worse than – 20 dB
